# P-405. A Case Against Universal *Candida auris* Screening

**DOI:** 10.1093/ofid/ofae631.606

**Published:** 2025-01-29

**Authors:** Palak Patel, Eric Roessler, Nicole Perez Vasquez, Molly Steele, Saba Rezaeian, Kristy Hill, Emily Landon, Patricia D Zuccaro

**Affiliations:** The University of Chicago, Chicago, Illinois; University of Chicago, Chicago, Illinois; Advocate Illinois Masonic Medical Center, Chicago, Illinois; University of Chicago, Chicago, Illinois; University of Chicago Medical Center, Chicago, Illinois; University of Chicago Medical Center, Chicago, Illinois; The University of Chicago Medicine, Chicago, Illinois

## Abstract

**Background:**

*Candida auris (C. auris)* is an emerging multidrug resistant pathogen that is prevalent in congregate living facilities like skilled nursing facilities (SNFs) and long term acute care hospitals (LTACHs). CDC recommends that individuals who recently resided in SNFs or LTACHs undergo active surveillance for *C. auris* colonization on admission to an acute care hospital.

**Figure:**
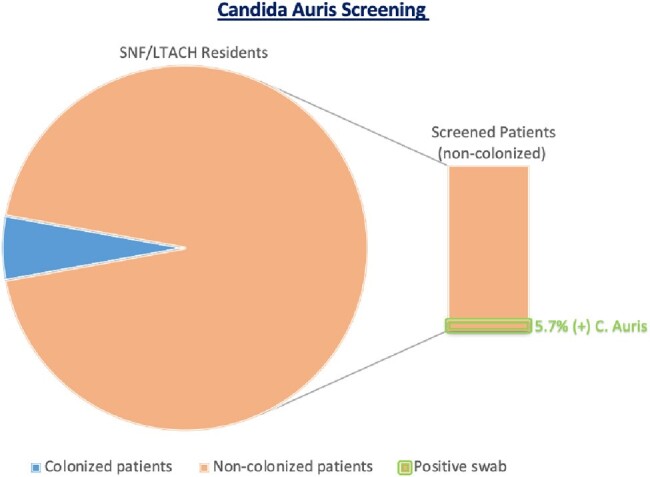

**Methods:**

University of Chicago Medicine is an 800 bed tertiary academic medical center. To assess feasibility of *C. auris* screening, UCM piloted active surveillance for high risk patients in the 24 bed medical intensive care unit (MICU). During the study period, 6/26/2023 to 4/3/2024, charge nurses screened newly admitted patients each shift and notified provider teams to order a *C. auris* screening for anyone who had previously resided in a SNF or LTACH. The screening test, a combined axilla and groin swab, was collected from each eligible patient and sent for PCR assay to Mayo Clinic Laboratories (Rochester, MN). Patients known to be *C. auris* colonized were not retested. The infection prevention and control team audited MICU admissions to ensure patients were not missed.

**Results:**

398 patients were admitted to the MICU and 94 were identified as high risk (previously in SNF or LTACH). Of these, 6 were already known to be *C. auris* colonized and were not tested. 88 total patients were swabbed for C. auris and 5 were positive, representing 5.7% of screened individuals. Turnaround time for this sendout lab was 4-5 days on average. During this time period, 4 patients in MICU developed a *C. auris* infection. Of those 4 patients only 1 was screened on admission and had a negative screen.

**Conclusion:**

While the burden of swabbing high risk patients was low, the burden of screening all admissions to identify those who were high risk was not. This strain on our workforce, coupled with the unexpectedly low rate of colonization, has prevented expansion of the program. Additionally, the long turnaround time for the screen requires either isolating all high risk patients for a large part of their hospital stay or enduring significant transmission risk if forgoing isolation, while awaiting screening results.

**Disclosures:**

**All Authors**: No reported disclosures

